# Enhanced Triplet Energy Transfer in Quantum Dot–Molecule Hybrids by Driving Bright State Redistribution

**DOI:** 10.1021/acs.nanolett.5c04652

**Published:** 2025-11-10

**Authors:** Peng Zhu, Xinze Liu, Meilin Guo, Qi Li, Guangxiong Hu, Jianbo Gao, Ying Shi

**Affiliations:** † Institute of Atomic and Molecular Physics, 12510 Jilin University, Changchun, 130012, China; ‡ Department of Chemistry, 7497 Brock University, St. Catharines, Ontario L2S 3A1, Canada

**Keywords:** triplet energy transfer, quantum dot, temperature, luminescence, transient absorption spectroscopy

## Abstract

Optimizing triplet energy transfer (TET) performance between quantum dots (QDs) and molecules is crucial, because it enables efficient triplet sensitization with promising optoelectronic applications. Current strategies prioritize static parameters such as QD size or shell engineering but pay less attention to fine-structured bright–dark excitonic states inherent to QDs. Herein, we demonstrate a thermally driven bright–dark redistribution as the governing mechanism for TET enhancement in naphthalene-functionalized CdSe/ZnS QDs. Temperature-resolved spectroscopy resolves a photoluminescence splitting (∼18 meV) below 233 K due to reverse-TET-mediated dark exciton accumulation. Critically, thermally activated redistribution elevates the bright-state proportion from 33.9% to 49.1%, achieving a 4.2-fold increase in TET rate and enhancing efficiency from 26.9% to 73.3%. This work establishes exciton engineering as a strategic approach for TET optimization in QD–molecular hybrids and provides fundamental insights into advanced photonic and energy conversion technologies.

## Graphical abstract

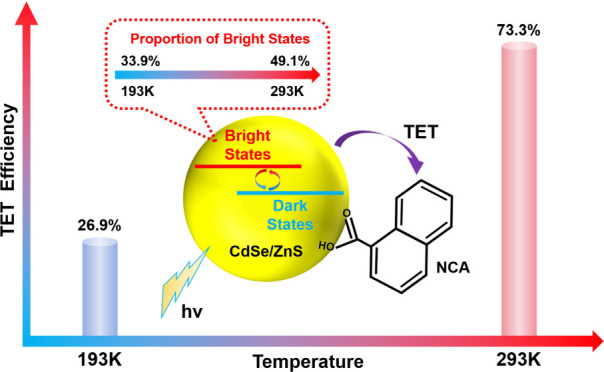

The triplet state of organic molecules, characterized by long lifetimes and high chemical reactivity, is the basis for emerging technologies in photon up-conversion and photodynamic therapy.^1–4^ Traditionally, the generation of this state has relied on sensitization through noble metal complexes, such as Ir, Pt, or Ru, and polycyclic aromatic hydrocarbons (PAHs).^5–8^ These methods have intrinsic limitations such as narrow absorption bands, high energy losses, and poor photostability, constraining energy collection and conversion. In recent years, leveraging the properties of quantum dots (QDs), including tunable bandgap,^9–11^ excellent photostability,^12,13^ and small energy difference between bright–dark states,^14,15^ the QD–organic molecule hybrid systems offer a powerful platform for triplet energy harvesting.^16–18^ Central to this functionality is triplet energy transfer (TET) between QDs and molecules, which enables ultrafast high-yield triplet sensitization. So far, the diverse combinations of QDs, including CdSe,^18,19^ InP,^20^ PbS/CdS,^21^ ZnSe/ZnS,^22^ and CsPbBr_3_ perovskite,^23,24^ with acceptor molecules such as PAHs,^18–24^ rhodamine,^25,26^ and functionalized oligothiophenes^27,28^ have been extensively investigated for TET optimization.

Current strategies for enhancing TET performance primarily focus on static design parameters. Dimensional tuning of QDs systematically modulates their bandgap energy and thus adjusts the donor–acceptor energy shift, achieving high-efficiency TET.^19,29,30^ Additionally, precise regulation of the QD–molecule triplet energy gap to less than 200 meV facilitates thermally activated delayed photoluminescence (TADPL) via reverse-TET (rTET).^19,20,24^ Engineering shell structures further suppresses trap states or competitive charge transfer pathways, thereby enhancing TET.^21,31^ However, these strategies based on static design fundamentally overlook a critical intrinsic property of QDs, which is the excitonic fine structure within QDs. This property arises from electron–hole exchange and further splits band-edge excitons into bright and dark states with small energy separation (Δ).^14,15,32,33^ At a cryogenic temperature of 5 K, the dark state has been revealed to participate in TET processes, while their inherently slower TET rates and extreme experimental conditions significantly constrain the application.^28^ Thermally driving bright–dark exchange offers a dynamic solution, which enables bright-exciton repopulation to enhance TET efficiency. This approach still encounters two principal challenges: (1) the thermal energy near room temperature (∼26 meV) exceeding typical bright–dark splitting energies (Δ < 10 meV),^32,34^ masking state-specific contributions, and (2) competing temperature-activated processes (e.g., defect trapping) that disturb TET dynamics.^31^

In this work, we constructed a TET system using CdSe/ZnS QDs (with a bright–dark state splitting energy level of Δ = 18 meV) as the donor and 1-naphthoic acid (NCA) molecules as the acceptor, respectively. In the range of 193–293 K, the TET kinetics were systematically investigated by temperature-dependent steady-state and transient absorption (TA) spectroscopy techniques. Notably, a low-temperature photoluminescence (PL) splitting phenomenon was observed, arising from the interplay between thermal effects and rTET. Within the applied temperature range, it facilitates the resolution of bright–dark state energy splitting at low temperatures as well as the dynamic modulation of bright-exciton populations via thermal redistribution. Critically, the dynamic analysis of TA spectra explicitly excludes interference from surface trap states. Further investigation into the temperature dependence of key TET parameters revealed that thermal activation substantially increases the proportion of bright excitons, which in turn leads to a notable enhancement in the TET efficiency and a several-fold acceleration of the TET rate.

The hybrid system under investigation is illustrated schematically in Scheme 1. This illustration displays the TET-related photophysical processes between donors and acceptors along with the corresponding energy levels. The QD–NCA complexes dispersed in hexane were prepared through a ligand exchange procedure; the details can be found in the Supporting Information (SI) Section SI1. The carboxyl group of the NCA molecule enables it to be effectively anchored to the QD surface by displacing native ligands, thus facilitating the required donor–acceptor electron orbital overlap for efficient TET. Figure 1a shows the UV–vis absorption and PL spectra of CdSe/ZnS QDs, NCA molecules, and QD–NCA complexes at room temperature. The pristine QDs exhibit a lowest exciton absorption peak at 454 nm (*E*_X_ ∼ 2.73 eV), and the NCA triplet energy (*E*_T_ ∼ 2.6 eV)^23,35^ creates a small energy shift (Δ*E* = 0.13 eV) between *E*_X_ and *E*_T_. Theoretically, this minor energy offset enables repopulation of QDs via thermally activated rTET following initial energy transfer to the acceptor.^19,24,36^ This process ultimately gives rise to a long-lived delayed emission. In addition, the QD–NCA complexes present strong absorption below 350 nm, while above this wavelength they maintain identical absorption features to pristine QDs. Fourier transform infrared spectroscopy (FTIR) spectra (Figure S2 in SI3) indicate that this strong absorption dominantly originates from surface-bound NCA molecules. Critically, the absence of spectral overlap between the NCA absorption spectra and the PL spectra of QDs precludes the possibility of Förster resonance energy transfer.^37,38^ Moreover, the absorption onset of the NCA lies at a substantially shorter wavelength than that of the QDs, which excludes PL quenching caused by energy transfer from QDs to the NCA singlet state and provides a basis for selective photoexcitation of QDs by a 400 nm pulse.

**Scheme 1:**
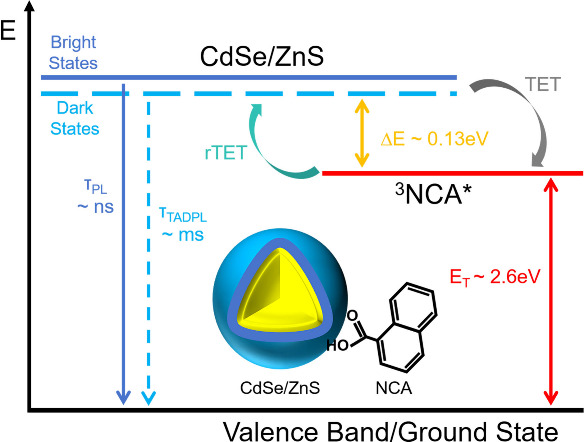
Energy Level Diagram and Photophysical Processes in the CdSe/ZnS-NCA Hybrid System

**Figure 1:**
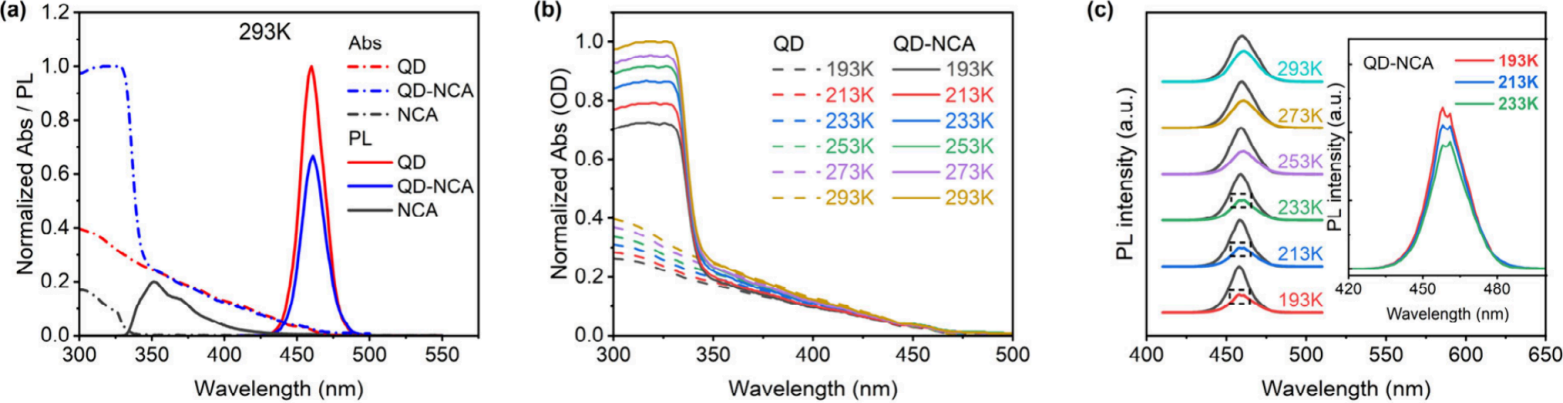
(a) The steady-state spectra of NCA molecules, CdSe/ZnS QDs, and QD–NCA complexes in hexane at room temperature. The PL spectra of QDs and QD–NCA complexes were tested by a 400 nm pulse, while that of NCA molecules was tested under 280 nm excitation. Temperature-dependent (b) absorption spectra and (c) PL spectra of CdSe/ZnS QDs and QD–NCA complexes (black for QDs and colors for QD–NCA complexes). The inset in (c) exhibits the PL splitting of the QD–NCA complex at low temperatures from 193 to 233 K.

Figure 1b and 1c illustrates the temperature-dependent absorption and PL spectra for the QDs and their NCA complexes. Based on the absorption spectral data, we quantified the number of NCA molecules adsorbed to QDs, giving a final ratio of ∼57 molecules per QD. This ratio can be considered to be constant across the temperature range studied (see SI1 for details). As the temperature decreased from 293 to 193 K, the quenching of QD emission by NCA was enhanced, with the quenching efficiency increasing from 33.2% to 66.0% (Table S1 in Section SI6). Notably, within the range of 193 to 233 K, a distinct PL splitting of ∼4 nm emerges in the QD–NCA complexes, as shown in Figure 1c, inset. Given the splitting is only in QD–NCA complexes while pristine QDs maintain a single and symmetric PL peak at all temperatures, the possibility of inhomogeneous broadening or size distribution effects is ruled out. This phenomenon is attributed to the fine-structure splitting of excitonic states in QDs, arising predominantly from electron–hole exchange interactions.^14,15,39,40^ Owing to the quantum confinement effect and the breaking of lattice symmetry in quantum dots,^32,39^ the exciton energy levels are further split into a bright state and a dark state, with the energy range of these states extending from a few to tens of meV.^14^ Moreover, this fine structure is highly sensitive to the size of the QDs, as well as external factors such as temperature.^34,41^ The clear observation of this splitting not only provides direct evidence of excitonic state redistribution but also establishes a foundation for further elucidating the role of bright–dark equilibria in the TET process.

To quantitatively understand the origin of the spectral splitting, the bright–dark state energy difference was extracted as Δ = 18 meV from the inset in Figure 1c, corresponding to the observed ∼4 nm splitting. When the temperature is below 233 K, thermal energy becomes insufficient to promote dark excitons to the higher-energy bright state.^28,42^ As the temperature increases beyond this threshold, thermal excitation enables a gradual redistribution of excitons between the two states, eventually approaching thermal equilibrium. Furthermore, the rTET process in our system facilitates exciton repopulation in QDs via a minor energy offset between QDs and NCA molecules. Given that the dark state has a lower energy barrier than the bright state,^32,39^ rTET preferentially populates this state. At temperatures below 233 K, this preferential filling in combination with suppressed thermal activation results in the substantial accumulation of dark excitons. The subsequent radiative recombination of these dark excitons, which possess a distinct energy offset relative to the bright state, thus gives rise to a resolvable splitting in the PL spectra.

Temperature-dependent TA spectroscopy was systematically employed to probe exciton recombination kinetics in isolated CdSe/ZnS QDs and QD–NCA complexes (Figures S4 and S5 in Section SI4). As demonstrated in Figure 2a, b, d, and e, both systems present a prominent exciton bleach (XB) feature at 454 nm, attributed to state-filling effects of band-edge excitons.^40,43–45^ Notably, temperature-dependent XB decay kinetics reveal contrasting trends. Figure 2c shows the decay kinetics of free QDs were accelerated as the temperature decreases, which originates from the trapping of carriers by surface defects that suppresses slow recombination components.^46–48^ In contrast, the QD–NCA complexes display prolonged decay times in Figure 2f, consistent with the increasing proportion of long-lived dark excitons at lower temperatures.^42,49,50^ This discrepancy analysis establishes that low-temperature PL splitting in QD–NCA complexes arises from bright–dark exciton thermal redistribution rather than defect trapping. In addition, the position of the XB peak is slightly shifted, which is consistent with the steady-state spectra, reflecting thermal modulation of the QD bandgap.

**Figure 2:**
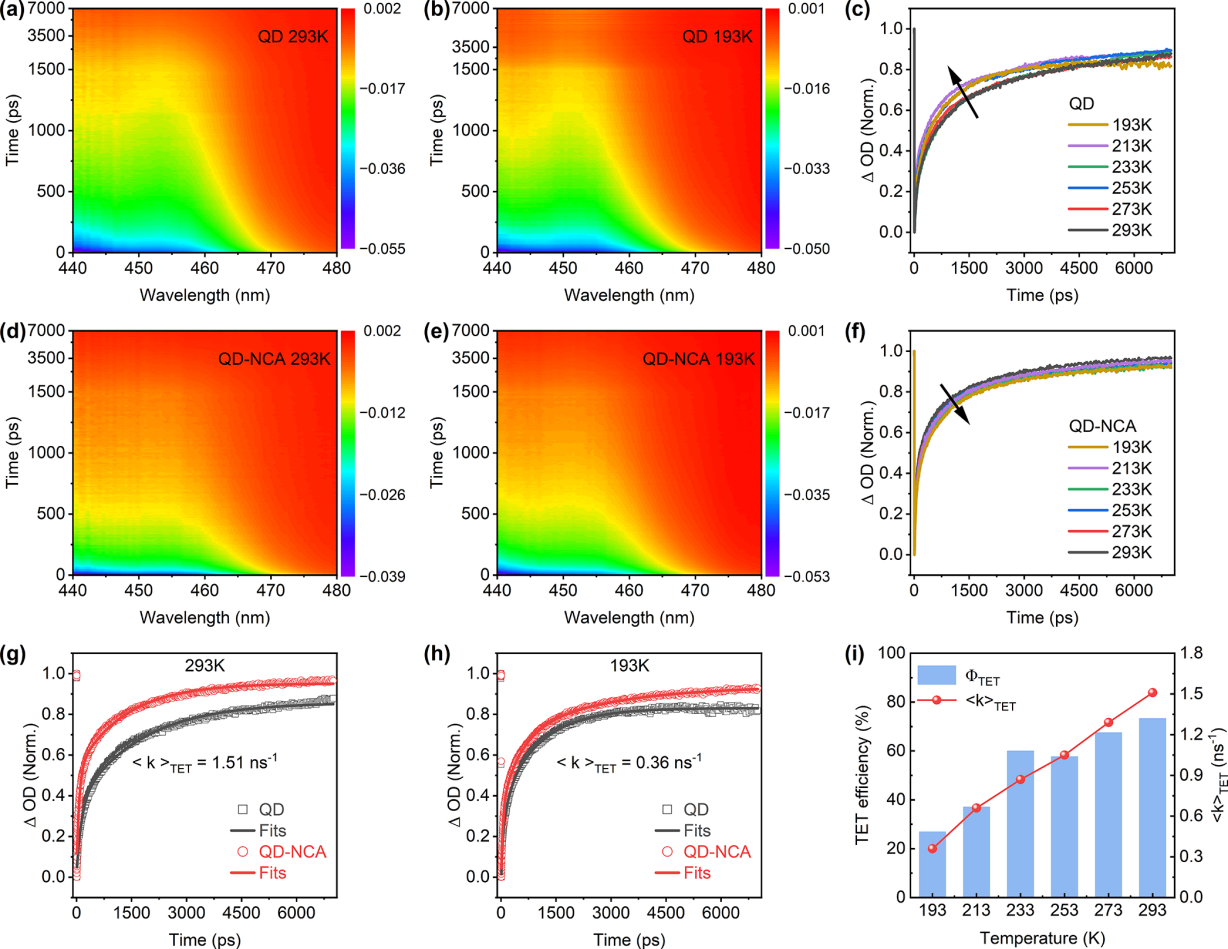
TA spectra of QDs at (a) 293 and (b) 193 K, recorded using a 400 nm pulse. (c) Temperature-dependent XB decay kinetics of QDs. (d–f) TA spectra and XB kinetics of QD–NCA complexes, similar to the plots in (a), (b), and (c). The XB recovery kinetics at (g) 293 K and (h) 193 K for pristine QDs (gray) and QD-NCA complexes (red) at the XB center. (i) Temperature-dependent TET rate and efficiency calculated from TA spectra.

In Figure 2g,h and Figure S6, comparative analysis of XB recovery kinetics shows a distinct acceleration in decay rates for QD–NCA complexes compared to isolated QDs, which directly reflects the TET-mediated exciton quenching. This acceleration exhibits pronounced thermal activation, as demonstrated by the progressive enhancement of TET rates across the studied temperature regime. To quantify TET dynamics, we applied a stretched-exponential function to fit the kinetic traces (see SI6 for further details).^18,51,52^ The calculated TET rate constants increase systematically with temperature, yielding a thermally activated TET rate enhancement from 0.36 ns^–1^ at 193 K to 1.51 ns^–1^ at 293 K, while TET efficiencies increased concurrently from 26.9% to 73.3% (Figure 2i). This increase in TET rate with temperature can be described qualitatively by Marcus theory:^17,23,53^

1 $$k_{TET}=\frac{2\pi }{\hbar }\frac{{\left|V\right|}^{2}}{\sqrt{4\pi \lambda k_{B}T}}\exp\left(-\frac{{\left(\lambda +\Delta G\right)}^{2}}{4\lambda k_{B}T}\right)$$

As shown in eq 1, there are three main parameters that truly determine the TET process: the reorganization energy (λ), the donor–acceptor driving force (Δ*E* = −Δ*G*), and the electron coupling strength (*V*). While the observed temperature dependence can be qualitatively described by an Arrhenius-type exponential term, a complete mechanistic interpretation requires a discussion of decoupling the temperature effects on λ, Δ*E*, and *V*. Concurrently, the resolvable PL splitting in QD–NCA complexes at low temperatures (Figure 1c, inset) and the temperature-dependent XB decay kinetics suggest an additional layer of complexity, which is the thermal redistribution between bright and dark exciton states.

To further confirm the existence of the rTET process, temperature-dependent PL lifetime measurements were employed to quantify TADPL dynamics across 193–293 K (Figure 3a, more details in SI7). Isolated QDs present a monotonic increase in PL lifetime from 6.37 ns at 193 K to 10.88 ns at 293 K, which trend is consistent with temperature-dependent XB decay kinetics in Figure 2c and can be attributed to thermal activation of shallow trap states.^46–48^ Conversely, the QD–NCA complexes demonstrate an opposite trend, from 30.79 ms at 193 K to 11.22 ms at 293 K. This divergence arises from the thermally activated nature of rTET. At low temperatures, the suppressed rTET retards the reverse exciton repopulation of QDs.^20,24,52^ Most notably, the delayed emission in QD–NCA complexes exhibits a six-order-of-magnitude lifetime enhancement relative to pristine QDs, which is directly visualized by the transition from nanosecond-scale to millisecond-scale lifetimes in Figure 3a. This directly provides evidence of efficient triplet-state involvement and rTET-mediated delayed emission.

**Figure 3:**
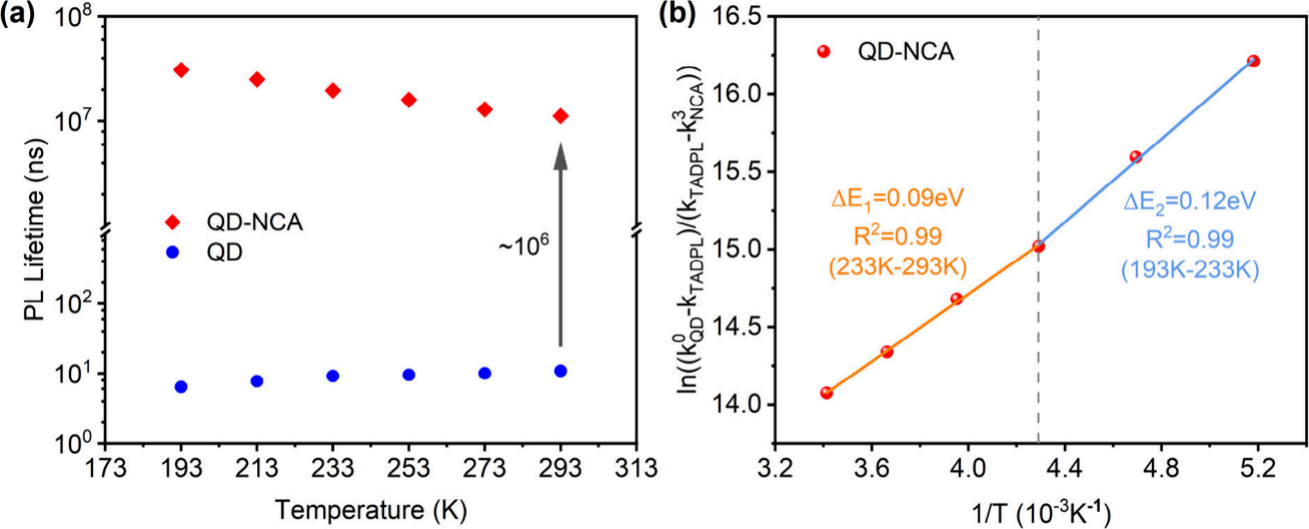
(a) Temperature-dependent PL lifetimes of QD and QD–NCA measured at 460 nm. (b) Variation of the energy gap (Δ*E*) fitted by the Boltzmann model.

The temperature-dependent decay kinetics of TADPL were analyzed using a modified Boltzmann equation, eq 2, to quantify the relationship between temperature and energy gap (Figure 3b):^20,36,54^

2 $$k_{TADPL}=\frac{k_{NCA}^{3}+k_{QD}^{0}\times \exp\left(-\frac{\Delta E}{k_{B}T}\right)}{1+\exp\left(-\frac{\Delta E}{k_{B}T}\right)}$$

This model unifies the TADPL rate (*k*_*TADPL*_ = 1/⟨τ⟩_*TADPL*_) with the energy gap (Δ*E*) and intrinsic rate constants of the QDs (*k*_*QD*_^0^ = 1/⟨τ⟩_*QD*_) and the NCA triplet state (*k*_*NCA*_^3^ = 1/⟨τ⟩_*NCA*_^3^). For model parametrization, the triplet lifetime of NCA was fixed to 55 ms, a value taken from prior literature.^20,55^ Fitting results revealed a clear temperature-dependent discrepancy in the energy gap. Between 193 and 233 K, the derived energy gap measured 0.12 eV, showing excellent agreement with the theoretical prediction of ∼0.13 eV. Conversely, at higher temperatures ranging from 233 to 293 K, the fitted energy gap contracted to 0.09 eV. This thermal modulation of the energy parameter reflects the modulating effect of temperature on the TET driving force.

For the reorganization energy of the TET process, it can be divided into two parts, inner (*λ*_*i*_) and outer (λ_*o*_) components.^56,57^ *λ*_*i*_ originates from the vibration of the molecule itself, while λ_*o*_ arises from the reorganization of the solvent or the surrounding medium. In the QD–molecular system, *λ*_*i*_ is determined by the molecule, since the molecular vibrational coupling is demonstrably stronger relative to the electron–phonon coupling within QDs. Here, *λ*_*i*_ of NCA was established at 0.32 eV, which is half of the TET coupling value between naphthalene molecules,^58^ while λ_*o*_ is assumed to be zero given the nonpolar hexane solvent.^59^ The rigid naphthalene core and nonpolar environment further ensure that λ remains essentially constant over the temperature range studied. According to eq 1 and the condition that the total λ exceeds the TET driving force (Δ*E* = 0.13 eV), the system remains firmly within the Marcus normal regime, ruling out inversion-region effects. Furthermore, it is estimated that from 193 to 293 K, when Δ*E* decreases from 0.12 to 0.09 eV, the increase in TET rate predicted by eq 1 is less than 50%. This value is much lower than the experimentally observed 4.2-fold increase in Figure 2i. Consequently, the significant TET rate enhancement cannot be explained by minor variations in the driving force.

The electronic coupling exhibits pronounced sensitivity to QD dimensions.^35,59–61^ To assess whether thermally induced lattice expansion could modulate this coupling via wave function overlap,^62^ we analyzed the temperature-dependent shifts of the lowest exciton absorption and PL peaks in both isolated QDs and QD–NCA complexes (Figure 4a). The near-perfect overlap of the shift trajectories for both samples confirms that the electronic structure of QDs is unaffected by surface-adsorbed NCA molecules. The observed parallel shift of ∼0.01 eV in the excitonic peaks, which can be well fitted by the Varshni model (see SI8 for details),^63^ indicates a slight bandgap narrowing due to lattice thermal expansion. This energy shift corresponds to a negligible dimensional change of ∼0.04 nm, as calculated through application of the quantum confinement effect (see SI2 for details). The initial QD dimensions were approximately 8.9 nm, as shown in Figure S1. Based on the size–wave function–TET rate model established by Wu and co-workers,^35,59^ this minimal structural variation, less than 0.5% in size, implies that temperature-induced variations in electronic coupling are insufficient to account for the enhanced TET rates.

**Figure 4:**
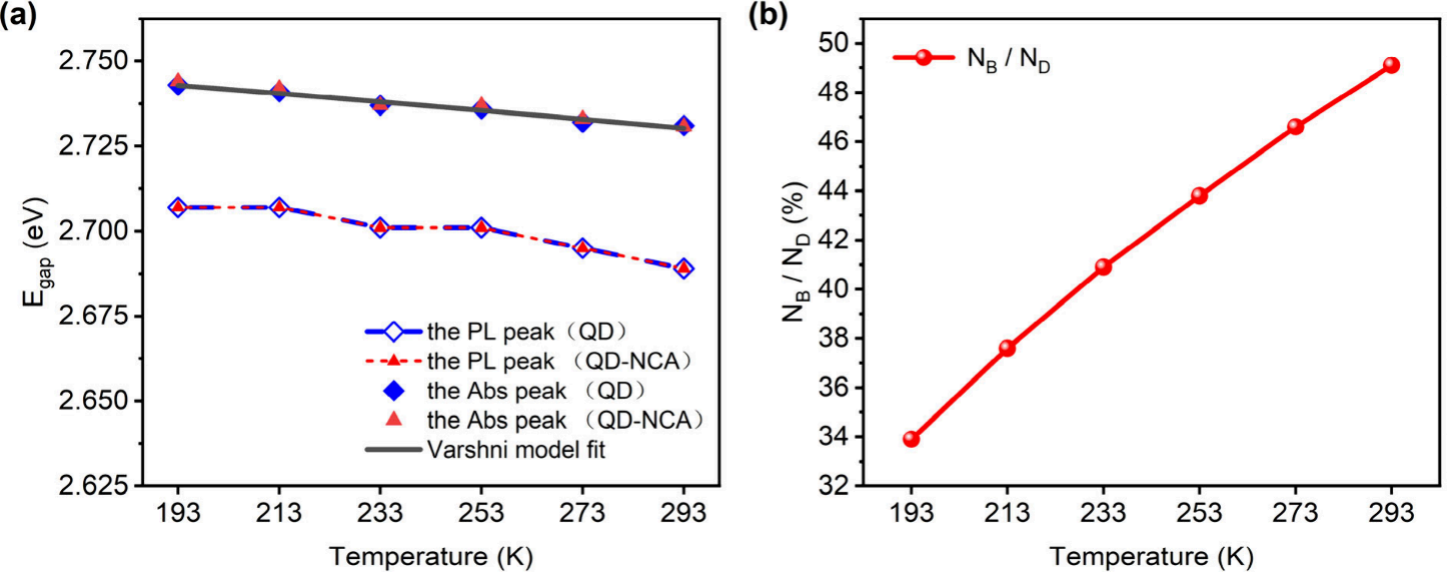
(a) Temperature dependence of the lowest exciton absorption and PL peak positions of the QDs extracted from absorption and fluorescence spectra. (b) Proportion of the bright–dark state varying with temperature.

Having established that the temperature has a negligible effect on other intrinsic parameters governing TET, we quantitatively evaluate the contribution of bright and dark exciton states in CdSe/ZnS QDs. In the exciton fine structure, bright and dark states exhibit not only energy splitting but also fundamental differences in their spin configurations and wave function properties.^16,32^ For Dexter-type TET, the bright state’s spin configuration highly matches with the molecular triplet acceptor, leading to a strong coupling. In contrast, the dark state’s wave function symmetry results in a significantly weaker coupling. Consequently, the bright state inherently serves as a more efficient triplet energy donor. Research has confirmed that dark excitons can also participate in the TET process, but their energy transfer rates are significantly lower than that of bright states.^28^ For isolated QDs, the bright and dark states can be assumed at thermal equilibrium and described by the Arrhenius model:^28,49^

3 $$\frac{N_{B}}{N_{D}}=e^{-\Delta /k_{B}T}$$

where *N*_*B*_ (bright state) and *N*_*D*_ (dark state) represent the respective exciton populations; Δ = 18 meV denotes the bright–dark splitting energy obtained from Figure 1c. Figure 4b demonstrates that increasing the temperature over the 100 K range elevates the bright-state proportion from 33.9% to 49.1%. This bright–dark redistribution induced by the temperature plays a dominant role in TET dynamics. Furthermore, we constructed eq 4 to qualitatively integrate this excitonic thermal equilibrium with the Marcus theory.

4 $${\left|V_{eff}\right|}^{2}\left(T\right)=N_{B}\cdot {\left|V_{B}\right|}^{2}+N_{D}\cdot {\left|V_{D}\right|}^{2}$$

where *V*_*eff*_ is the effective electronic coupling, and *V*_*B*_ and *V*_*D*_ represent the electronic coupling of the bright and dark states, respectively. Here, temperature is not altering the intrinsic coupling of individual exciton states but rather dynamically modulating the ratio of bright to dark states in the initial state. This, in turn, influences the effective spin configuration of the system and favors specific reaction pathways. As a result, the electronic coupling is no longer a static parameter but a temperature-dependent parameter. Consequently, the thermally enhanced bright-state population drives a 4.2-fold increase in effective TET rates from 0.36 to 1.51 ns^–1^ and a concurrent rise in efficiency from 26.9% to 73.3%. These findings clearly identify excitonic thermal redistribution between bright and dark states as the dominant mechanism for TET enhancement, establishing exciton population control as a crucial strategy for optimizing QD–molecular hybrid systems.

In summary, we have demonstrated that the thermal redistribution of bright and dark excitons serves as a dominant mechanism for enhancing TET in QD–molecular hybrid systems. Through temperature-dependent steady-state and TA spectroscopy, we identified a clear spectral signature of rTET-mediated dark exciton accumulation below 233 K, and TA kinetic analysis confirmed the exclusion of defect trapping pathways. With increasing temperature, the thermal repopulation of bright excitons drives a 4.2-fold enhancement in the TET rate and an efficiency increase from 26.9% to 73.3%. Marcus theory analysis rules out contributions from driving force changes or QD size variations, which strongly support exciton thermal redistribution as the primary mechanism. Furthermore, the system exhibits a six-order-of-magnitude enhancement in delayed emission arising from efficient rTET. This work establishes thermal exciton management as a powerful strategy for controlling energy transfer processes, providing fundamental insights for advanced photonic and energy conversion technologies.

## Supplementary Material



## Notes

Materials and experimental methods; characterization of CdSe/ZnS quantum dots; temperature-dependent transient absorption spectra; fitting of temperature-dependent kinetics; temperature-dependent PL lifetime curves and fitting results (PDF)

## References

[1] Schulze T. F., Schmidt T. W. (2015). Photochemical Upconversion: Present Status and Prospects
for Its Application to Solar Energy Conversion. Energy Environ. Sci..

[2] Singh-Rachford T. N., Castellano F. N. (2010). Photon Upconversion Based on Sensitized Triplet-Triplet
Annihilation. Coord. Chem. Rev..

[3] Li X., Kolemen S., Yoon J., Akkaya E. U. (2017). Activatable Photosensitizers:
Agents for Selective Photodynamic Therapy. Adv.
Funct. Mater..

[4] DeRosa M. (2002). Photosensitized
Singlet Oxygen and Its Applications. Coord.
Chem. Rev..

[5] Zhao J., Wu W., Sun J., Guo S. (2013). Triplet Photosensitizers:
From Molecular
Design to Applications. Chem. Soc. Rev..

[6] Pun A. B., Sanders S. N., Sfeir M. Y., Campos L. M., Congreve D. N. (2019). Annihilator
Dimers Enhance Triplet Fusion Upconversion. Chem. Sci..

[7] Sun J., Wu W., Zhao J. (2012). Long-Lived
Room-Temperature Deep-Red-Emissive Intraligand
Triplet Excited State of Naphthalimide in Cyclometalated Ir^III^ Complexes and Its Application in Triplet-Triplet Annihilation-Based
Upconversion. Chem.—Eur. J..

[8] Ji S., Guo H., Wu W., Wu W., Zhao J. (2011). Ruthenium­(II) Polyimine-Coumarin
Dyad with Non-emissive ^3^IL Excited State as Sensitizer
for Triplet-Triplet Annihilation Based Upconversion. Angew. Chem., Int. Ed..

[9] Murray C. B., Norris D. J., Bawendi M. G. (1993). Synthesis and Characterization of
Nearly Monodisperse CdE (E = Sulfur, Selenium, Tellurium) Semiconductor
Nanocrystallites. J. Am. Chem. Soc..

[10] Akkerman Q. A., D’Innocenzo V., Accornero S., Scarpellini A., Petrozza A., Prato M., Manna L. (2015). Tuning the Optical
Properties of Cesium Lead Halide Perovskite Nanocrystals by Anion
Exchange Reactions. J. Am. Chem. Soc..

[11] Cai T., Wang J., Li W., Hills-Kimball K., Yang H., Nagaoka Y., Yuan Y., Zia R., Chen O. (2020). Mn^2+^ /Yb^3+^ Codoped CsPbCl_3_ Perovskite
Nanocrystals with Triple-Wavelength Emission for Luminescent Solar
Concentrators. Adv. Sci..

[12] Chen Y., Vela J., Htoon H., Casson J. L., Werder D. J., Bussian D. A., Klimov V. I., Hollingsworth J. A. (2008). “Giant”
Multishell CdSe Nanocrystal Quantum Dots with Suppressed Blinking. J. Am. Chem. Soc..

[13] Hills-Kimball K., Yang H., Cai T., Wang J., Chen O. (2021). Recent Advances
in Ligand Design and Engineering in Lead Halide Perovskite Nanocrystals. Adv. Sci..

[14] Efros Al. L., Rosen M., Kuno M., Nirmal M., Norris D. J., Bawendi M. (1996). Band-Edge Exciton in Quantum Dots of Semiconductors
with a Degenerate Valence Band: Dark and Bright Exciton States. Phys. Rev. B.

[15] Scholes G. D., Rumbles G. (2006). Excitons in Nanoscale Systems. Nat. Mater..

[16] Wang J., Ding T., Nie C., Wang M., Zhou P., Wu K. (2020). Spin-Controlled Charge-Recombination Pathways across the Inorganic/Organic
Interface. J. Am. Chem. Soc..

[17] Huang Z., Tang M. L. (2017). Designing Transmitter
Ligands That Mediate Energy Transfer
between Semiconductor Nanocrystals and Molecules. J. Am. Chem. Soc..

[18] Mongin C., Garakyaraghi S., Razgoniaeva N., Zamkov M., Castellano F. N. (2016). Direct
Observation of Triplet Energy Transfer from Semiconductor Nanocrystals. Science.

[19] La
Rosa M., Denisov S. A., Jonusauskas G., McClenaghan N. D., Credi A. (2018). Designed Long-Lived Emission from CdSe Quantum Dots through Reversible
Electronic Energy Transfer with a Surface-Bound Chromophore. Angew. Chem., Int. Ed..

[20] Zhang X., Hudson M. H., Castellano F. N. (2022). Engineering Long-Lived Blue Photoluminescence
from InP Quantum Dots Using Isomers of Naphthoic Acid. J. Am. Chem. Soc..

[21] Huang Z., Xu Z., Mahboub M., Li X., Taylor J. W., Harman W. H., Lian T., Tang M. L. (2017). PbS/CdS Core-Shell Quantum Dots Suppress
Charge Transfer and Enhance Triplet Transfer. Angew. Chem., Int. Ed..

[22] Lin X., Chen Z., Han Y., Nie C., Xia P., He S., Li J., Wu K. (2022). ZnSe/ZnS Core/Shell Quantum Dots
as Triplet Sensitizers toward Visible-to-Ultraviolet B Photon Upconversion. ACS Energy Lett..

[23] Luo X., Han Y., Chen Z., Li Y., Liang G., Liu X., Ding T., Nie C., Wang M., Castellano F. N., Wu K. (2020). Mechanisms of Triplet Energy Transfer across the Inorganic Nanocrystal/Organic
Molecule Interface. Nat. Commun..

[24] He S., Han Y., Guo J., Wu K. (2021). Long-Lived Delayed Emission from
CsPbBr_3_ Perovskite Nanocrystals for Enhanced Photochemical
Reactivity. ACS Energy Lett..

[25] DuBose J. T., Kamat P. V. (2021). Directing Energy
Transfer in Halide Perovskite-Chromophore
Hybrid Assemblies. J. Am. Chem. Soc..

[26] Qin C., Li J., Song J., Ma S., Zhang J., Jia G., Jiao Z., Zhu Z., Jiang Y., Zhou Z. (2023). Ultrafast
Energy Transfer from Perovskite Quantum Dots to Chromophore. Results Phys..

[27] Xu Z., Jin T., Huang Y., Mulla K., Evangelista F. A., Egap E., Lian T. (2019). Direct Triplet Sensitization of Oligothiophene
by Quantum Dots. Chem. Sci..

[28] Jin T., He S., Zhu Y., Egap E., Lian T. (2022). Bright State Sensitized
Triplet Energy Transfer from Quantum Dot to Molecular Acceptor Revealed
by Temperature Dependent Energy Transfer Dynamics. Nano Lett..

[29] Wang L., Mu P., Zhou Z., Zhang X., Liu D., Liang Y., Liang G. (2023). Ultrafast Spectroscopy of Size-Dependent
Hole-Transfer-Mediated Triplet
Energy Transfer in a Quantum Dot-Molecule Complex. Results Phys..

[30] Luo S., Zhang Y., Zhu Y., Wang X. J., Ran X., He Y., Kuang Y., Chi Z., Guo L. (2023). Size-Regulated Hole
and Triplet Energy Transfer from CdSe Quantum Dots to Organic Acceptors
for Enhancing Singlet Oxygen Generation. Inorg.
Chem..

[31] Huang Z., Xia P., Megerdich N., Fishman D. A., Vullev V. I., Tang M. L. (2018). ZnS Shells
Enhance Triplet Energy Transfer from CdSe Nanocrystals for Photon
Upconversion. ACS Photonics.

[32] Sercel P. C., Efros A. L. (2018). Band-Edge Exciton
in CdSe and Other II-VI and III-V
Compound Semiconductor Nanocrystals - Revisited. Nano Lett..

[33] Nirmal M., Norris D. J., Kuno M., Bawendi M. G., Efros Al. L., Rosen M. (1995). Observation of the “Dark Exciton” in CdSe Quantum Dots. Phys. Rev. Lett..

[34] Bornschlegl A. J., Lichtenegger M. F., Luber L., Lampe C., Bodnarchuk M. I., Kovalenko M. V., Urban A. S. (2024). Dark-Bright Exciton
Splitting Dominates
Low-Temperature Diffusion in Halide Perovskite Nanocrystal Assemblies. Adv. Energy Mater..

[35] Han Y., Luo X., Lai R., Li Y., Liang G., Wu K. (2019). Visible-Light-Driven
Sensitization of Naphthalene Triplets Using Quantum-Confined CsPbBr_3_ Nanocrystals. J. Phys. Chem. Lett..

[36] Yonemoto D. T., Papa C. M., Mongin C., Castellano F. N. (2020). Thermally
Activated Delayed Photoluminescence: Deterministic Control of Excited-State
Decay. J. Am. Chem. Soc..

[37] Clapp A. R., Medintz I. L., Mattoussi H. (2006). Förster
Resonance Energy Transfer
Investigations Using Quantum-Dot Fluorophores. ChemPhysChem.

[38] Lin S., Keskar G., Wu Y., Wang X., Mount A. S., Klaine S. J., Moore J. M., Rao A. M., Ke P. C. (2006). Detection
of Phospholipid-Carbon Nanotube Translocation Using Fluorescence Energy
Transfer. Appl. Phys. Lett..

[39] Becker M. A., Vaxenburg R., Nedelcu G., Sercel P. C., Shabaev A., Mehl M. J., Michopoulos J. G., Lambrakos S. G., Bernstein N., Lyons J. L., Stöferle T., Mahrt R. F., Kovalenko M. V., Norris D. J., Rainò G., Efros A. L. (2018). Bright Triplet Excitons in Caesium Lead Halide Perovskites. Nature.

[40] Klimov V. I. (2007). Spectral
and Dynamical Properties of Multiexcitons in Semiconductor Nanocrystals. Annu. Rev. Phys. Chem..

[41] Han Y., Liang W., Lin X., Li Y., Sun F., Zhang F., Sercel P. C., Wu K. (2022). Lattice Distortion
Inducing Exciton Splitting and Coherent Quantum Beating in CsPbI3
Perovskite Quantum Dots. Nat. Mater..

[42] Gaponenko M. S., Lutich A. A., Tolstik N. A., Onushchenko A. A., Malyarevich A. M., Petrov E. P., Yumashev K. V. (2010). Temperature-Dependent
Photoluminescence of PbS Quantum Dots in Glass: Evidence of Exciton
State Splitting and Carrier Trapping. Phys.
Rev. B.

[43] Huang J., Huang Z., Yang Y., Zhu H., Lian T. (2010). Multiple Exciton
Dissociation in CdSe Quantum Dots by Ultrafast Electron Transfer to
Adsorbed Methylene Blue. J. Am. Chem. Soc..

[44] Zhu H., Song N., Lian T. (2010). Controlling
Charge Separation and
Recombination Rates in CdSe/ZnS Type I Core-Shell Quantum Dots by
Shell Thicknesses. J. Am. Chem. Soc..

[45] Lim J. W. M., Guo Y., Feng M., Cai R., Sum T. C. (2024). Making
and Breaking of Exciton Cooling Bottlenecks in Halide Perovskite Nanocrystals. J. Am. Chem. Soc..

[46] Wu J., Chen L., Zhao Y., Xiong Z., Ji W., Lei Y. (2021). Temperature-Dependent Recombination Dynamics and Electroluminescence
Characteristics of Colloidal CdSe/ZnS Core/Shell Quantum Dots. Appl. Phys. Lett..

[47] Javaux C., Mahler B., Dubertret B., Shabaev A., Rodina A. V., Efros Al. L., Yakovlev D. R., Liu F., Bayer M., Camps G., Biadala L., Buil S., Quelin X., Hermier J. P. (2013). Thermal Activation of Non-Radiative
Auger Recombination
in Charged Colloidal Nanocrystals. Nat. Nanotechnol..

[48] Zhang J., Zhang X., Zhang J. Y. (2010). Dependence
of Microstructure and
Luminescence on Shell Layers in Colloidal CdSe/CdS Core/Shell Nanocrystals. J. Phys. Chem. C.

[49] De
Mello Donegá C., Bode M., Meijerink A. (2006). Size- and
Temperature-Dependence of Exciton Lifetimes in CdSe Quantum Dots. Phys. Rev. B.

[50] Crooker S. A., Barrick T., Hollingsworth J. A., Klimov V. I. (2003). Multiple Temperature
Regimes of Radiative Decay in CdSe Nanocrystal Quantum Dots: Intrinsic
Limits to the Dark-Exciton Lifetime. Appl. Phys.
Lett..

[51] Luo X., Lai R., Li Y., Han Y., Liang G., Liu X., Ding T., Wang J., Wu K. (2019). Triplet Energy Transfer
from CsPbBr_3_ Nanocrystals Enabled by Quantum Confinement. J. Am. Chem. Soc..

[52] Mongin C., Moroz P., Zamkov M., Castellano F. N. (2018). Thermally
Activated Delayed Photoluminescence from Pyrenyl-Functionalized CdSe
Quantum Dots. Nat. Chem..

[53] Wang J., Ding T., Gao K., Wang L., Zhou P., Wu K. (2021). Marcus Inverted Region
of Charge Transfer from Low-Dimensional Semiconductor
Materials. Nat. Commun..

[54] Yonemoto D. T., Papa C. M., Sheykhi S., Castellano F. N. (2021). Controlling
Thermally Activated Delayed Photoluminescence in CdSe Quantum Dots
through Triplet Acceptor Surface Coverage. J.
Phys. Chem. Lett..

[55] Gahlaut R., Joshi H. C., Joshi N. K., Pandey N., Arora P., Rautela R., Suyal K., Pant S. (2013). Luminescence
Characteristics
and Room Temperature Phosphorescence of Naphthoic Acids in Polymers. J. Lumin..

[56] Marcus R. A. (1993). Electron
Transfer Reactions in Chemistry. Theory and Experiment. Rev. Mod. Phys..

[57] Brédas J. L., Beljonne D., Coropceanu V., Cornil J. (2004). Charge-Transfer and
Energy-Transfer Processes in π-Conjugated Oligomers and Polymers:
A Molecular Picture. Chem. Rev..

[58] Yost S. R., Hontz E., Yeganeh S., Van Voorhis T. (2012). Triplet vs
Singlet Energy Transfer in Organic Semiconductors: The Tortoise and
the Hare. J. Phys. Chem. C.

[59] Lai R., Liu Y., Luo X., Chen L., Han Y., Lv M., Liang G., Chen J., Zhang C., Di D., Scholes G. D., Castellano F. N., Wu K. (2021). Shallow Distance-Dependent
Triplet Energy Migration Mediated by Endothermic Charge-Transfer. Nat. Commun..

[60] Li X., Huang Z., Zavala R., Tang M. L. (2016). Distance-Dependent
Triplet Energy Transfer between CdSe Nanocrystals and Surface Bound
Anthracene. J. Phys. Chem. Lett..

[61] Zhu H., Song N., Lian T. (2010). Controlling
Charge Separation and
Recombination Rates in CdSe/ZnS Type I Core-Shell Quantum Dots by
Shell Thicknesses. J. Am. Chem. Soc..

[62] Blaudeck T., Zenkevich E. I., Cichos F., Von Borczyskowski C. (2008). Probing Wave
Functions at Semiconductor Quantum-Dot Surfaces by Non-FRET Photoluminescence
Quenching. J. Phys. Chem. C.

[63] Varshni Y. P. (1967). Temperature
Dependence of the Energy Gap in Semiconductors. Physica.

