# Supplementary material for: Enhanced Triplet Energy Transfer in Quantum Dot–Molecule Hybrids by Driving Bright State Redistribution
Source: Nano Lett. 2025 Nov 10;25(46):16515–21. doi: 10.1021/acs.nanolett.5c04652 (PMC12637308; doi:10.1021/acs.nanolett.5c04652)
Supplement: Supplementary file 1 [file nl5c04652_si_001.pdf]

Supporting Information for:

Enhanced Triplet Energy Transfer in Quantum Dot-Molecule Hybrids by Driving Bright State Redistribution

*Peng Zhu[1], Xinze Liu[1], Meilin Guo[1], Qi Li[1], Guangxiong Hu[1], Jianbo Gao[2] and Ying Shi[1]**

[1]Institute of Atomic and Molecular Physics, Jilin University, Changchun, 130012, People's Republic of China

[2]Department of Chemistry, Brock University, St. Catharines, ON L2S 3A1, Canada

Table of Contents

SI1: Materials and Experimental Methods

SI2: Characterization of CdSe/ZnS core/shell Quantum Dots (QDs)

SI3: Evidence of ligand exchange

SI4: Temperature Dependent Transient Absorption (TA) Spectra of CdSe/CdS QDs and Naphthalene-Functionalized CdSe/ZnS QD Complexes (QD-NCA)

SI5: Comparison of Exciton Bleach (XB) of QDs and QD-NCA at Applied Temperatures

SI6: Fitting of Temperature Dependent XB Kinetics and Calculated Triplet Energy Transfer Parameters

SI7: Temperature Dependent PL Lifetime Curves and Fitting Results of QDs and QD-NCA

SI8: Fitting of Temperature Dependent Bandgap of QDs

SI1: Materials and Experimental Methods

Chemicals and solvents

The CdSe/ZnS core/shell QDs were purchased from Mesolight Inc. (Suzhou, China), with the surface ligands comprising oleic acid, oleylamine, and dodecanethiol. Hexane (98%, with molecular sieves) and 1-naphthoic acid (1-NCA, ≥98%) were purchased from Aladdin (China) and Macklin (China), respectively. All chemicals were used as received, without any further purification.

Preparation of CdSe/ZnS QD-NCA complexes

The QD-NCA complexes were prepared by adding 1-NCA powder into the CdSe/ZnS QD solution, followed by ultrasonic mixing for 30 minutes. The solution was then left undisturbed for 2 hours and filtered, resulting in a clear solution containing the QD-NCA complexes. Due to the negligible solubility of NCA in hexane, we believed that all NCA molecules in the solution after filtration were bound to the QD surface. Moreover, the final prepared QD-NCA complexes displayed long-term stability in hexane over an extended period, with no occurrence of desorption during the course of the experiment.

The average number of 1-NCA molecules attached to each QD was determined using UV-Vis absorption spectroscopy. According to the Lambert-Beer law, the NCA:QD ratio (n) can be expressed as:

$$n = \frac{A(NCA)/\varepsilon(NCA)}{A(QD)/\varepsilon(QD)} \tag{S1}$$

Where A and ε stand for absorbance and extinction coefficient, respectively. Based on the steady-state spectra, it can be inferred that the NCA:QD ratio (n) at room temperature is ~57. Furthermore, the relative ratio of acceptors to nanocrystals is correlated with the size of the QDs.[1,2] In our study, the size of the quantum dots remained largely constant despite variations in temperature, and the samples did not experience desorption during the experiment. Most importantly, no quantitative differences were observed in the transient behavior of these samples. Therefore, we postulate that the NCA:QD ratio at different temperatures is approximately the same and has no substantive impact on our conclusions.

Transmission electron microscopy (TEM) measurements

The samples were prepared by depositing CdSe/ZnS QDs dispersed in hexane onto a copper grid covered with an ultrathin carbon film. TEM images were subsequently obtained using a JEOL JEM-2100F TEM.

Steady-state measurements

Linear absorption spectra were measured with a UV3600IPLUS UV-visible spectrophotometer from Shimadzu. The photoluminescence (PL) spectra and lifetime were recorded by Edinburgh Instruments FLS980 and FLS920 spectrometers, respectively.

Femtosecond transient absorption (fs-TA) measurements

Details about femtosecond transient absorption spectroscopy have been discussed in previous literature.[3,4] Briefly, the output pulses from the femtosecond laser (Coherent Astrella, 800 nm, 35 fs, 2 mJ/pulses, and 1 kHz repetition rate) were split into two beams at a ratio of 9:1 using a beam splitter. One of the stronger beams passed through the BBO crystal and was frequency doubled to generate a 400 nm pump pulse. The other beam passed through the sapphire crystal and generated supercontinuous white light with a wavelength range of 440 to 700 nm as probe light. These two beams intersected at the sample with a small angle ($\theta \leq 5^{\circ}$), and the probe light was collected using a spectrometer (HELIOS). Single wavelength kinetic traces were analyzed using Origin 2024.

Temperature dependent measurements

In steady-state testing, the liquid nitrogen refrigeration system included with the equipment was used to provide a variable temperature environment for the samples. And for ultrafast experiments, the temperature of the samples was controlled using a Janis VPF-100 Series cryostat and a Model 22C cryogenic temperature controller.

The temperature range spanned from 193 K to 293 K, with increments of 20 K between each test point. The samples remain liquid in this temperature range (melting point of hexane = 178 K, boiling point of hexane = 341 K), thus avoiding problems related to phase transitions. The samples were placed in a closed quartz cuvette with an optical path length of 1 cm to prevent changes in sample concentration due to solvent evaporation. All tests were carried out under vacuum conditions.

SI2: Characterization of CdSe/ZnS core/shell Quantum Dots (QDs)

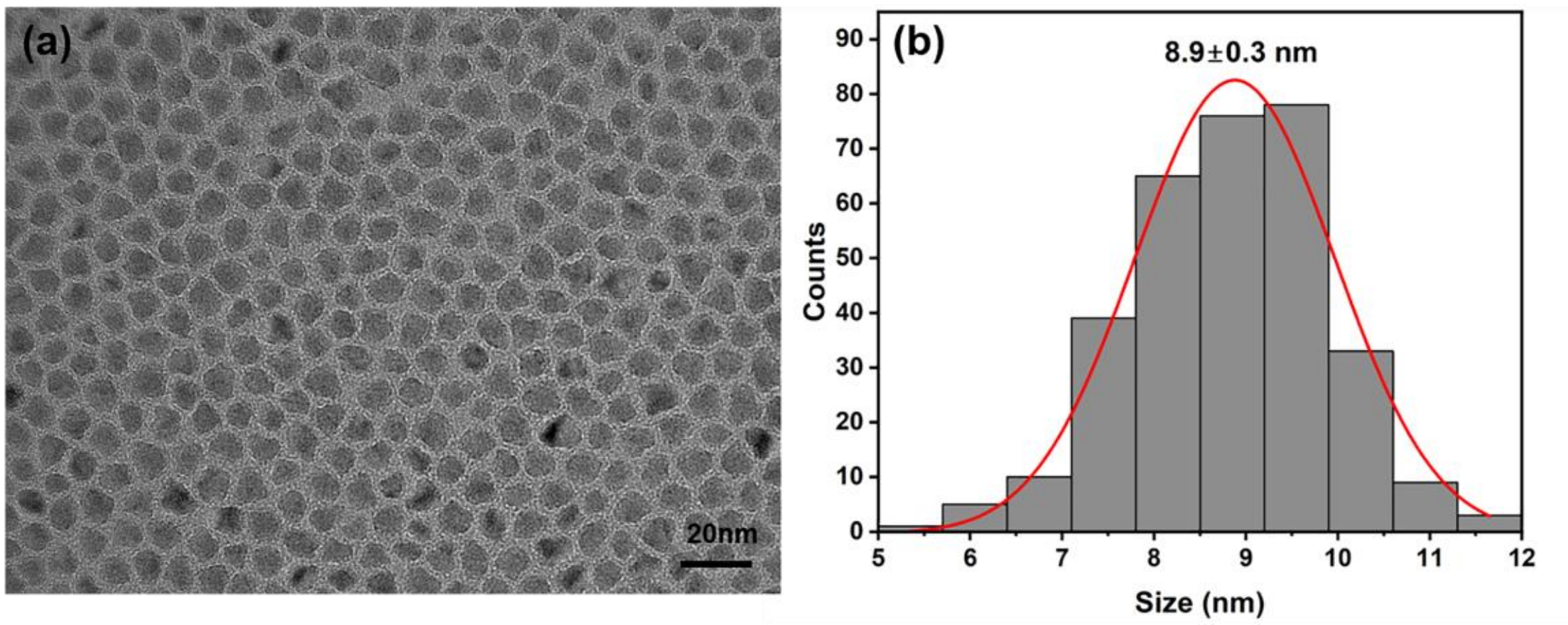

Figure S1. (a) Transmission Electron Microscopy (TEM) image and (b) Size distribution histograms of CdSe/ZnS core/shell QDs used in this study.

Calculations of QD size change

The Brus equation can be used to describe size-dependent energy changes in quantum-limited domain systems such as QDs.[5,6] In order to facilitate the ensuing discussion, the equation is simplified as follows: the bandgap energy is inversely proportional to the square of the radius of the quantum dot.

$$E_{QD} = E_{bulk} + \frac{h^2}{8R^2}\left(\frac{1}{m_e^*} + \frac{1}{m_h^*}\right) \quad (S2)$$

Where E_{bulk} is the band gap of the CdSe bulk material (~1.74 eV), R is the radius of the QDs, m_e^* and m_h^* are the effective masses of the electron and hole. The band gap variation relation can be expressed as：

$$E_1 = E_{bulk} + \frac{h^2}{8{R_1}^2}\left(\frac{1}{m_e^*} + \frac{1}{m_h^*}\right) \quad (S3)$$

$$E_2 = E_{bulk} + \frac{h^2}{8{R_2}^2}(\frac{1}{m_e^*} + \frac{1}{m_h^*}) \qquad \text{(S4)}$$

Further onwards, we get:

$$\frac{E_2 - E_{bulk}}{E_1 - E_{bulk}} = \frac{{R_1}^2}{{R_2}^2} \qquad \text{(S5)}$$

Based on the experimentally determined bandgap energies (E_1 = 2.73 eV, E_2 = 2.74 eV) and initial radius (R_1 = 4.45 nm) from Figure 2 and Figure S1, the derived thermal-induced size change ΔR = (0.02 ± 0.01) nm.

SI3: Evidence of ligand exchange

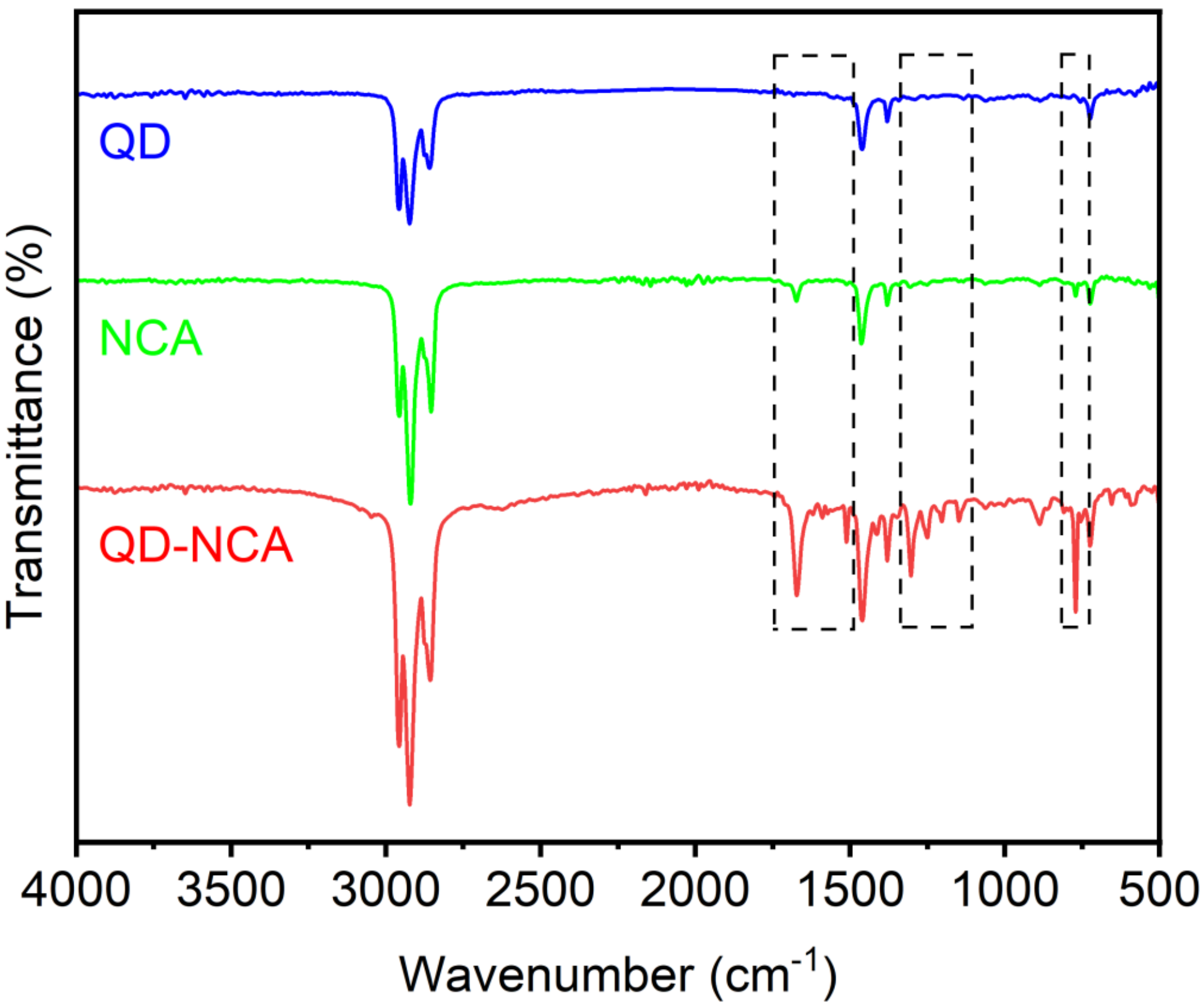

Figure S2. Fourier-transform infrared spectroscopy (FTIR) spectra of CdSe/ZnS QDs, free NCA molecules (prepared using the same steps as for the QD-NCA complexes) and QD-NCA complexes. Compared to the free NCA, new characteristic peaks appearing in the QD-NCA complexes are located at 1589.54 cm^{-1} and 1511.84 cm^{-1}, corresponding to carboxylate symmetric and asymmetric stretching vibrations,[7,8] clearly demonstrating chemical bonding between NCA carboxyl groups and the QD surface. And new peaks appearing at 1303.63 cm^{-1}, 1250.83 cm^{-1}, 1203.73 cm^{-1}, and 1148.78 cm^{-1} can be attributed to novel vibrational modes induced by intermolecular interactions after NCA molecules adsorb onto the quantum dot surface. Furthermore, compared to free NCA, the QD-NCA complex exhibits significantly enhanced peak

intensities at 1672.91 cm^{-1} and 771.48 cm^{-1}, indicating an increased density of NCA molecules on the QD surface.

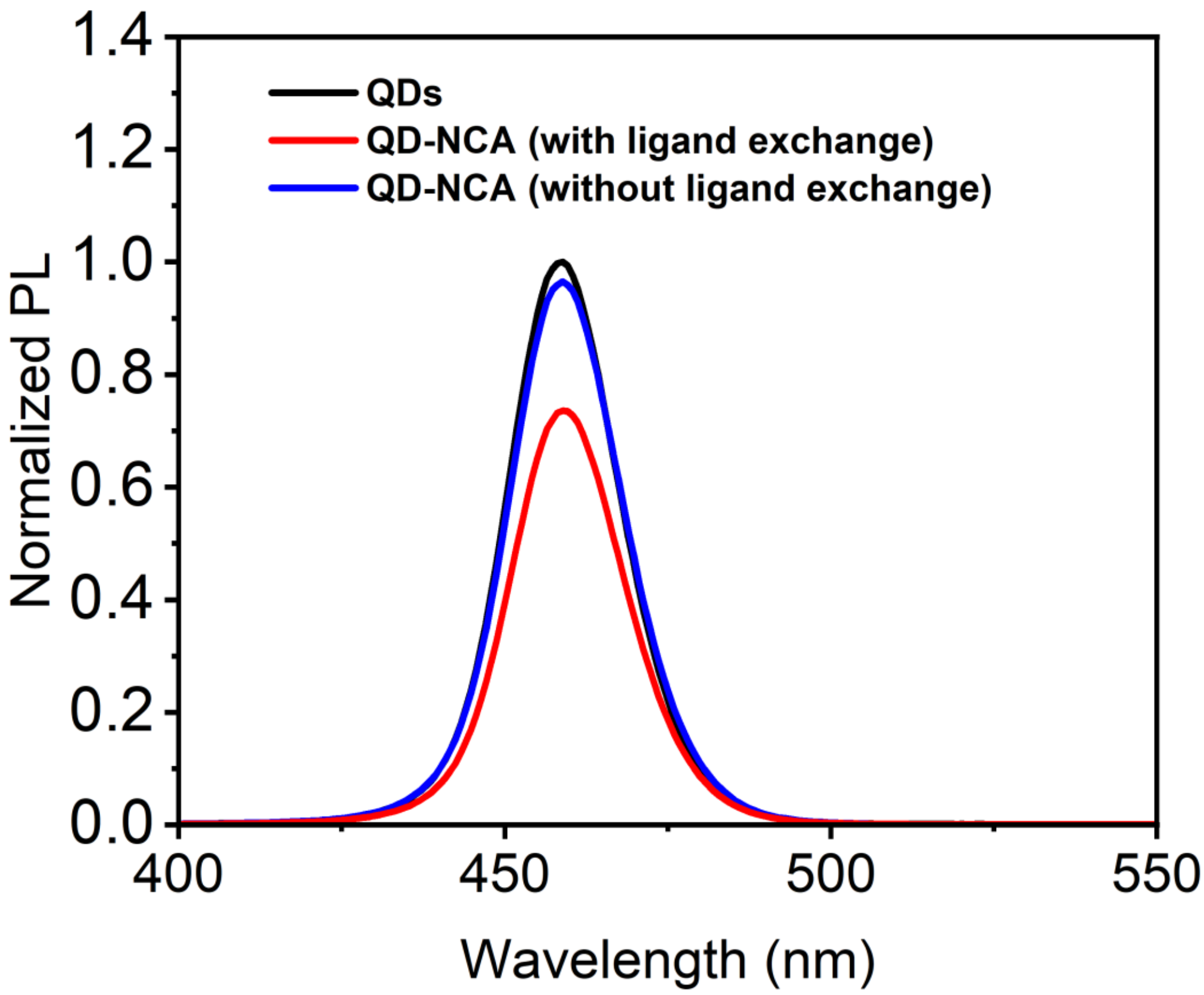

Figure S3. PL spectra of QD-NCA complexes (with and without ligand exchange). The control experiments demonstrate that no marked PL quenching was observed in the QD-NCA samples without ligand exchange. In contrast, fully ligand-exchanged QD-NCA complexes show significantly enhanced PL quenching, indicating successful binding of NCA molecules to the QD surface.

SI4: Temperature Dependent Transient Absorption (TA) Spectra of CdSe/CdS QDs and Naphthalene-Functionalized CdSe/ZnS QD Complex (QD-NCA)

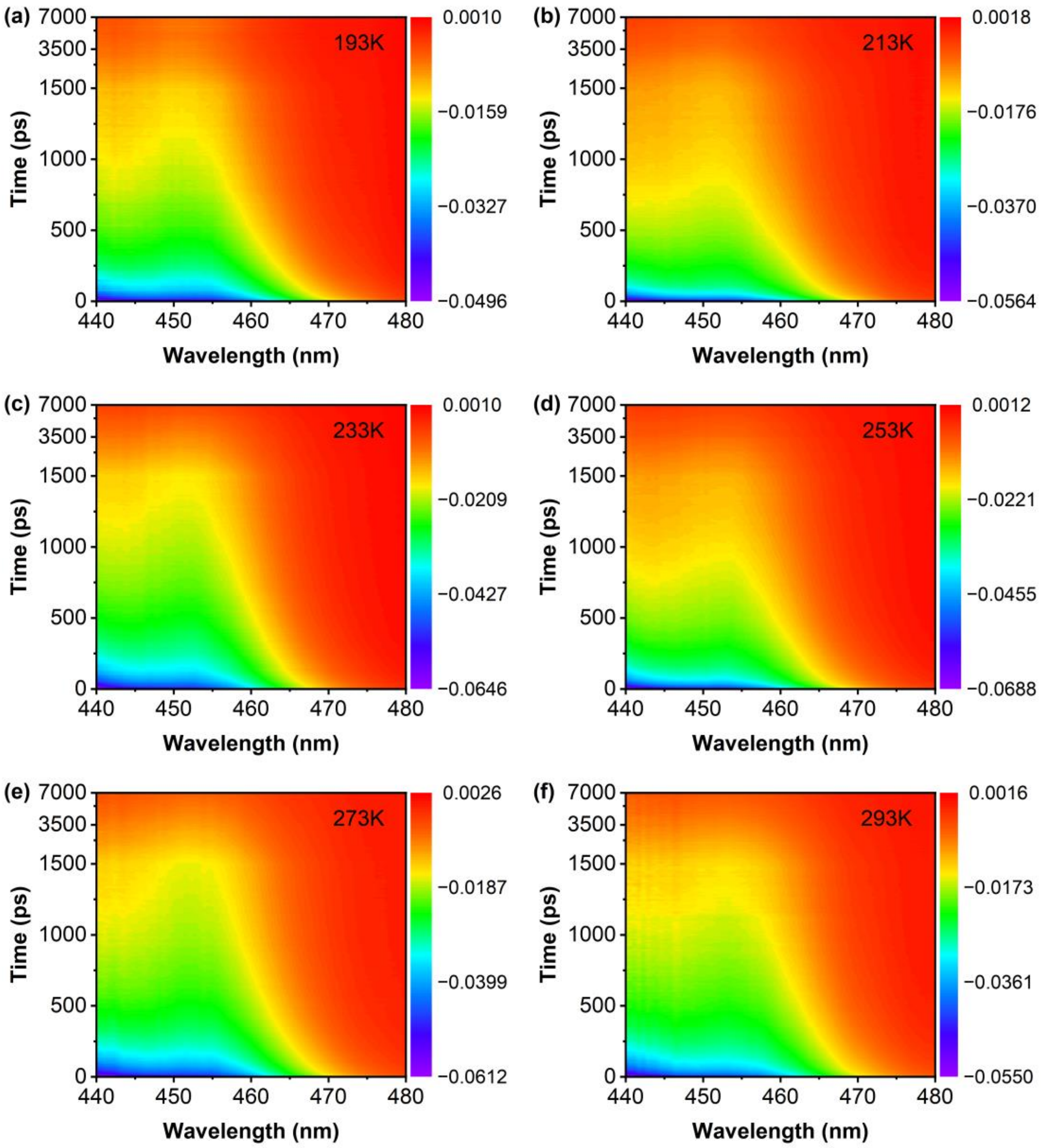

Figure S4. TA spectra of CdSe/ZnS core/shell QDs at different temperatures after excitation by a 400 nm pulse: (a) 193 K; (b) 213 K; (c) 233 K; (d) 253 K; (e) 273 K; (f) 293 K. (Where, the data is plotted in the range of 0-1500 ps using linear

coordinates and from 1500-7000 ps using logarithmic coordinates.)

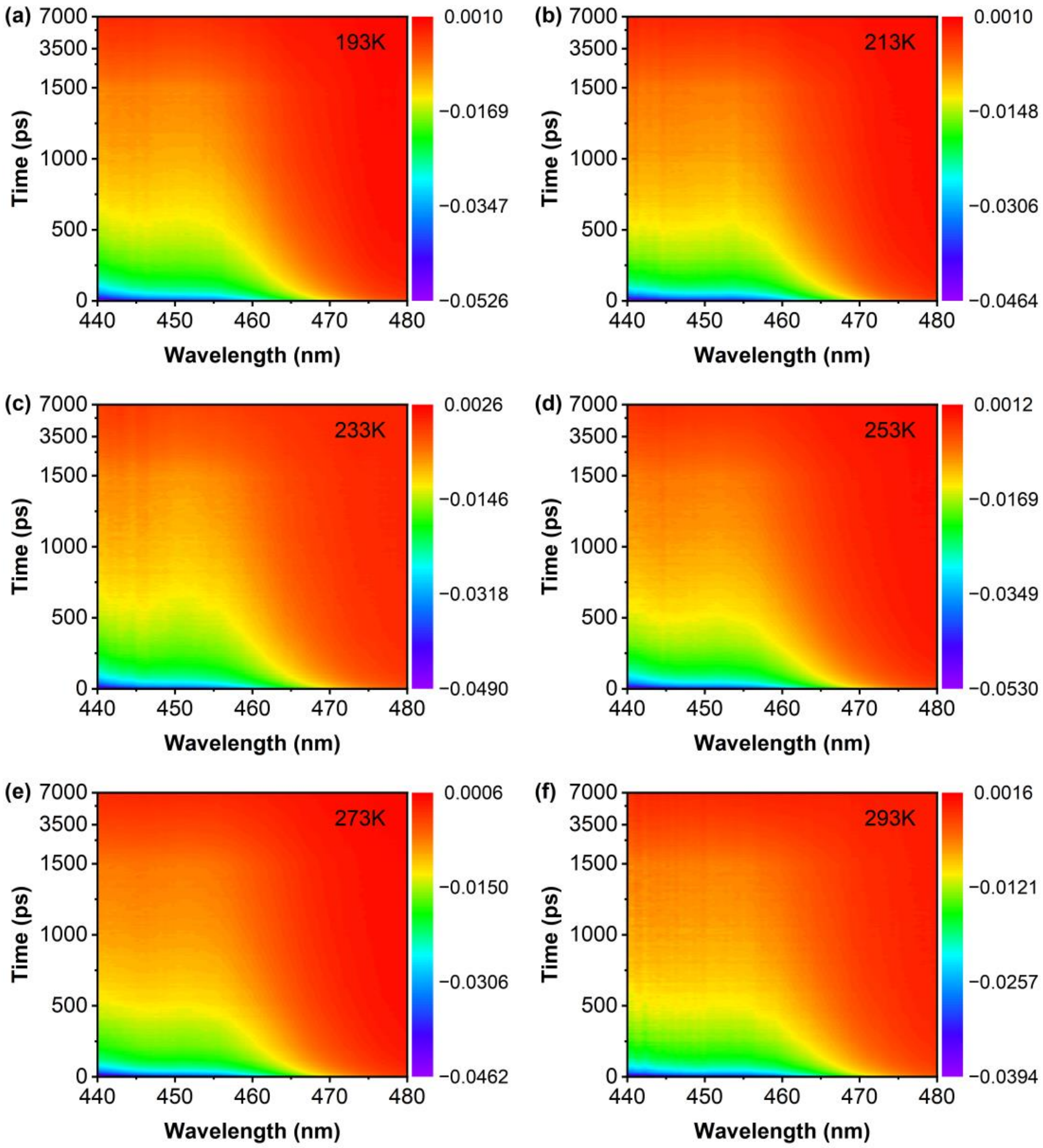

Figure S5. TA spectra of QD-NCA complexes at different temperatures after excitation by a 400 nm pulse: (a) 193 K; (b) 213 K; (c) 233 K; (d) 253 K; (e) 273 K; (f) 293 K. (Where, the data is plotted in the range of 0-1500 ps using linear coordinates and from 1500-7000 ps using logarithmic coordinates.)

SI5: Comparison of Exciton Bleach (XB) of QDs and QD-NCA at Applied Temperatures

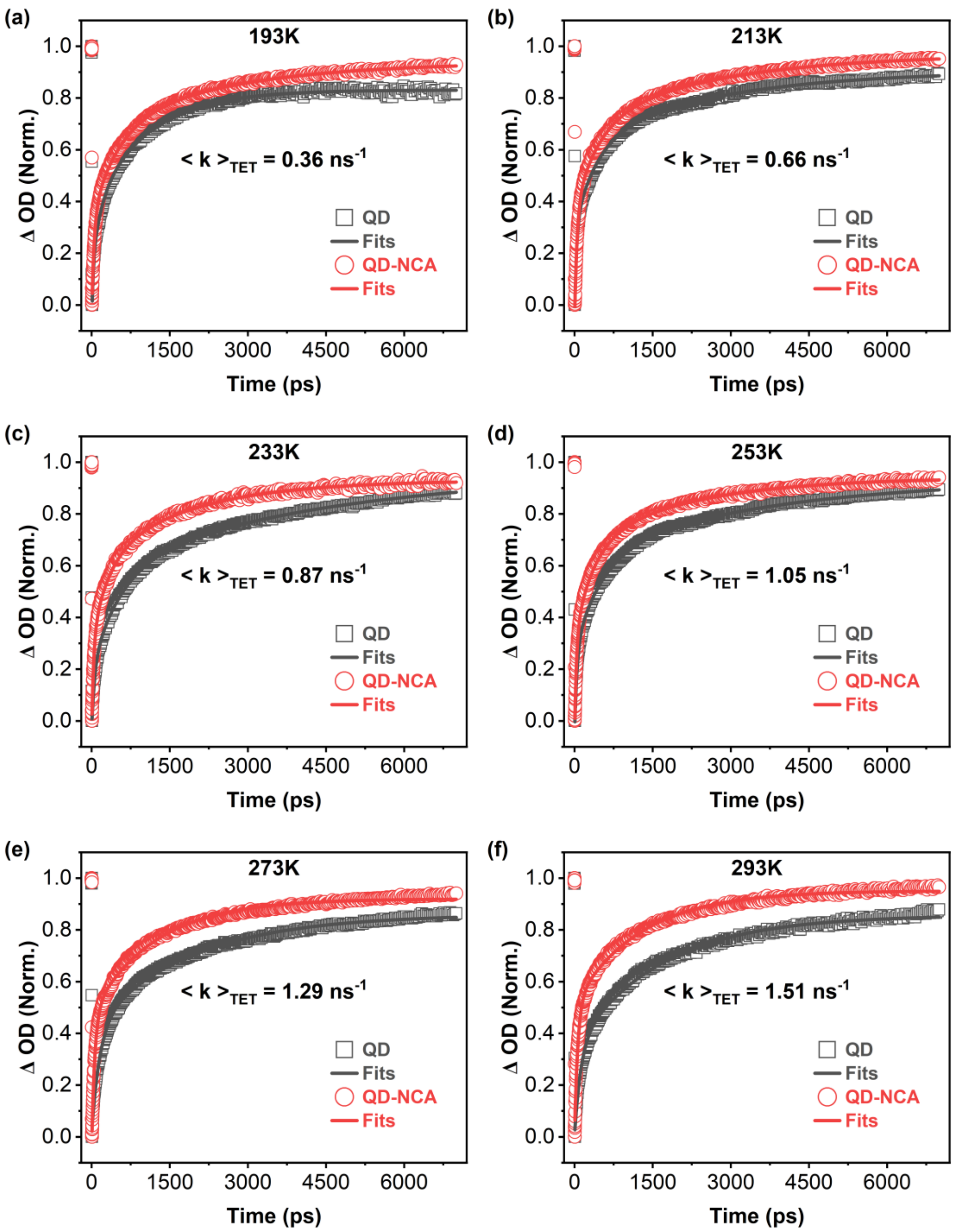

Figure S6. Temperature-dependent TA kinetics of QDs and QD-NCA complexes probed at the lowest exciton absorption peak of QDs (~454 nm) by a 400 nm pump

pulse. Temperatures range from (a) 193 K to (f) 293 K.

SI6: Fitting of Temperature Dependent XB Kinetics and Calculated Triplet Energy Transfer (TET) Parameters

Calculations of PL quenching efficiency

The PL quenching efficiency of QDs by NCA was calculated using the following formulae:

$$\varphi = 1 - \frac{I_{QD-NCA}}{I_{QD}} \quad (S6)$$

Where I_{QD-NCA} and I_{QD} are the PL intensities of QD-NCA complexes and free QDs, respectively, measured under the same conditions. The results of the calculations were shown in Table S1.

TA single wavelength kinetics fitting

Considering that the QD excited state and the NCA triplet acceptors could be influenced by temperature fluctuations, resulting in varying energy transfer rates, a stretched exponential function was employed to capture the rate of TET.[2,9,10] The kinetic curves derived from the transient absorption spectra were amenable to fitting using the following equations, and the results from these fitting procedures are displayed in Figure S4, and Table S1:

$$\Delta A = A_{TET} \times e^{-\left(\frac{t}{\tau_{TET}}\right)^{\beta}} \quad (S7)$$

Where A_{TET} and τ_{TET} are the amplitude and the stretched lifetime, respectively,

corresponding to the TET between CdSe/ZnS QDs and the surface-bound NCA molecules and β is the stretching exponent.

The average weighted lifetime $\langle\tau\rangle$ was calculated using the following equation with τ_{TET} and β values obtained from fitting:[11]

$$\langle\tau\rangle = \frac{\tau_{TET}}{\beta} \times \Gamma\left(\frac{1}{\beta}\right) = \frac{\tau_{TET}}{\beta} \times \int_0^{\infty} u^{\frac{1}{\beta}-1} e^{-u} du \tag{S8}$$

Thus, the rate constants of TET ($\langle k\rangle_{TET}$) from NC to PCA and the TET yields (Φ_{TET}) could be calculated from the following equations:

$$\langle k\rangle_{TET} = \langle k\rangle_{QD-NCA} - \langle k\rangle_{QD} = \frac{1}{\langle\tau\rangle_{QD-NCA}} - \frac{1}{\langle\tau\rangle_{QD}} \tag{S9}$$

$$\Phi_{TET} = \frac{\langle k\rangle_{TET}}{\langle k\rangle_{QD-NCA}} \tag{S10}$$

Where $\langle k\rangle_{QD-NCA}$ and $\langle k\rangle_{QD}$ are the average weighted lifetimes obtained from stretched exponential fitting of CdSe/ZnS exciton bleach with and without surface-anchored NCA acceptors, respectively.

Table S1. Triplet energy transfer parameters at different temperatures

T (K)	Samples	PL quenching Efficiency (%)	τ (ps)	β	$\langle \tau \rangle$ (ps)	$\langle k \rangle$ (ns^{-1})	$\langle k \rangle_{TET}$ (ns^{-1})	Φ_{TET} (%)
193	QD	-	250.37	0.2837	1018.93	0.98	0.36	26.9
	QD-NCA	61.0	278.99	0.3496	746.68	1.34		
213	QD	-	216.84	0.2825	889.65	1.12	0.66	37.1
	QD-NCA	59.2	186.58	0.3323	562.04	1.78		
233	QD	-	915.46	0.4102	1720.50	0.58	0.87	60.0
	QD-NCA	56.4	231.41	0.3325	687.64	1.45		
253	QD	-	446.65	0.3367	1292.98	0.77	1.05	57.7
	QD-NCA	50.6	159.03	0.3088	548.66	1.82		
273	QD	-	586.05	0.3458	1604.98	0.62	1.29	67.5
	QD-NCA	40.5	136.15	0.2924	522.87	1.91		
293	QD	-	823.46	0.3817	1822.96	0.55	1.51	73.3
	QD-NCA	33.2	188.46	0.3559	485.72	2.06		

SI7: Temperature Dependent PL Lifetime Curves and Fitting Results of QDs and QD-NCA

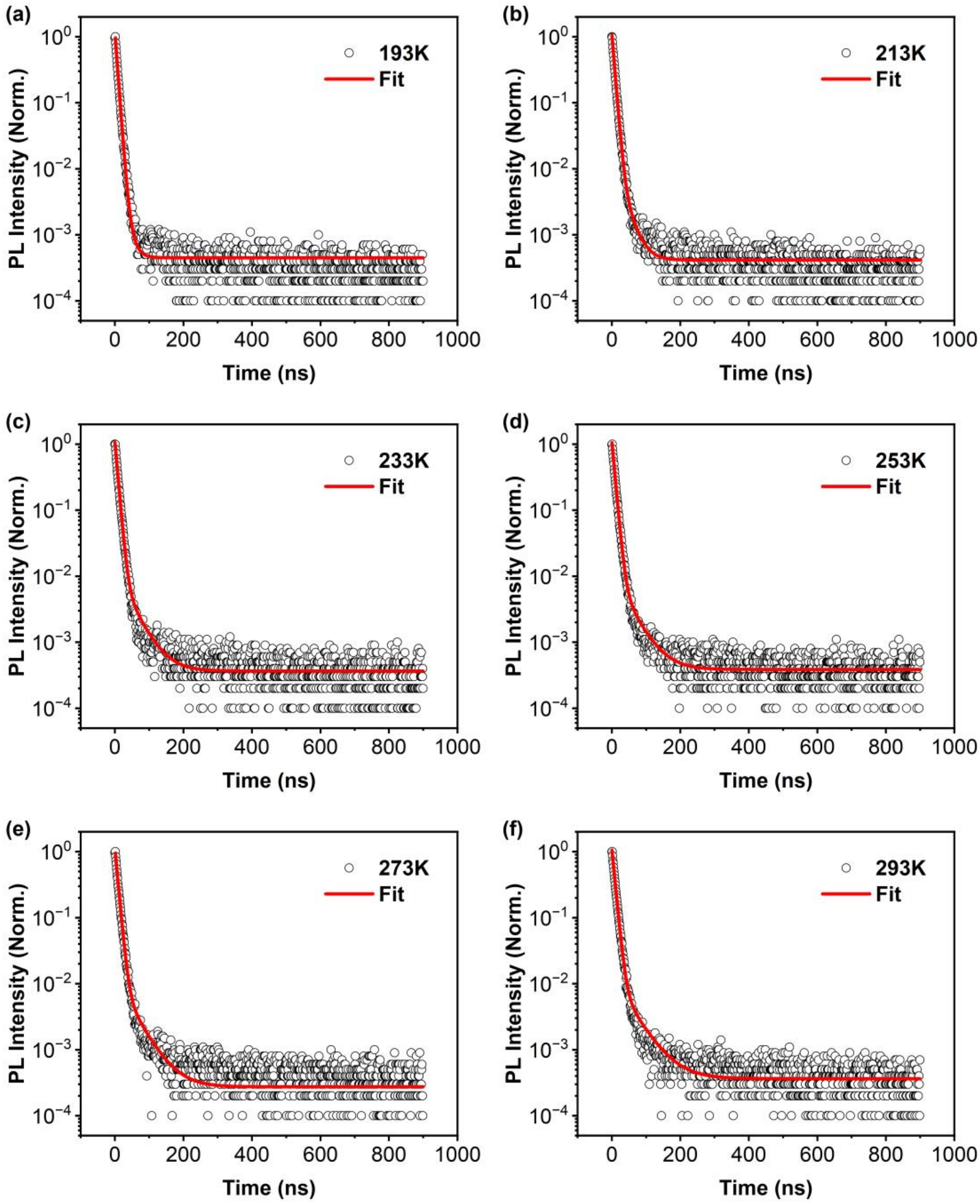

Figure S7. PL lifetime curves of QDs under 400 nm excitation at different temperatures: (a) 193 K; (b) 213 K; (c) 233 K; (d) 253 K; (e) 273 K; (f) 293 K; monitored at 460 nm, corresponding to the band-edge emission wavelength of QDs.

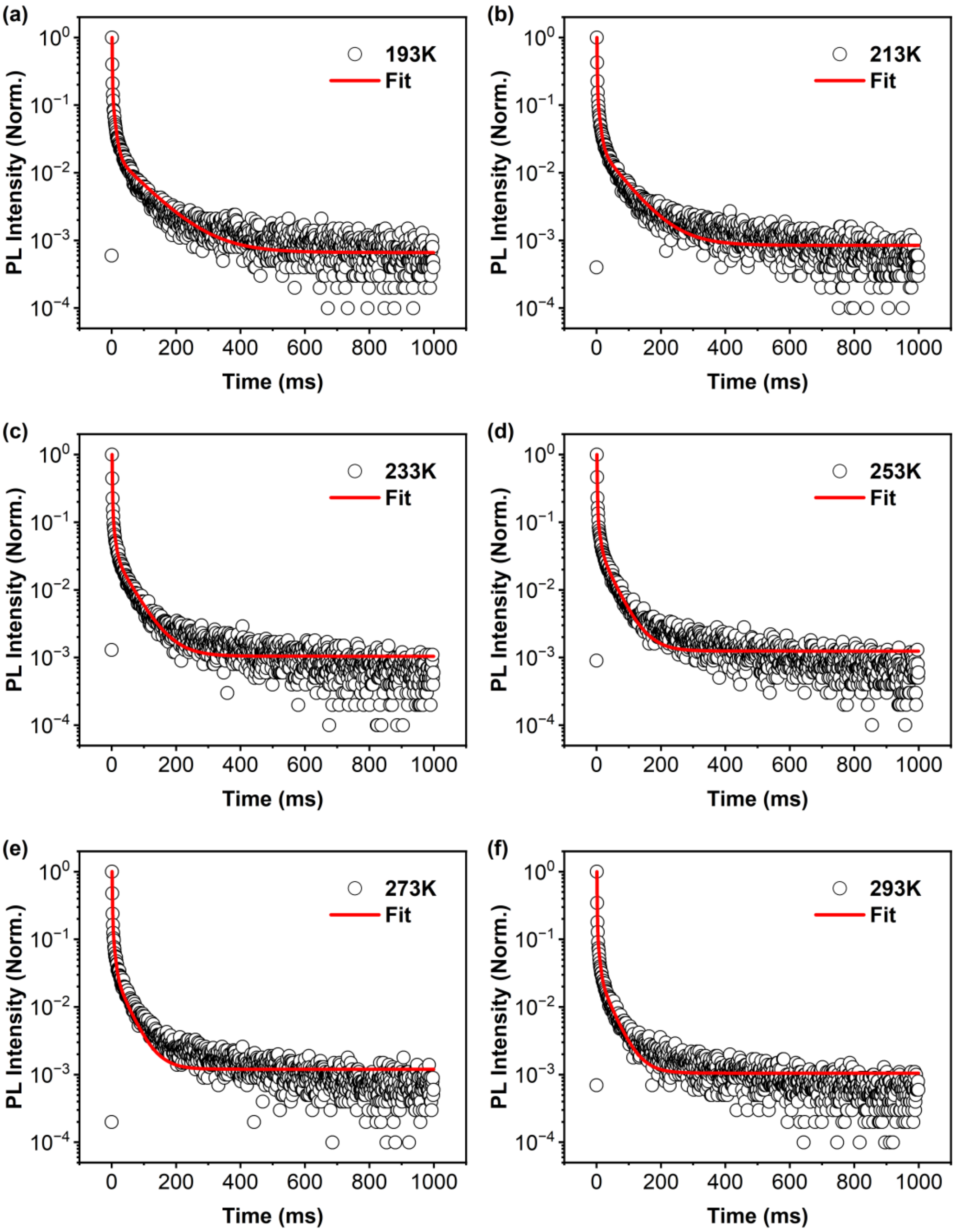

Figure S8. PL lifetime curves of QD-NCA complexes under 400 nm excitation at different temperatures: (a) 193 K; (b) 213 K; (c) 233 K; (d) 253 K; (e) 273 K; (f) 293 K; monitored at 460 nm, corresponding to the band-edge emission wavelength of QDs.

As shown in Figure 3a, when monitored at 460 nm—corresponding to the QD emission wavelength—the QD-NCA complexes exhibit a lifetime extension from nanoseconds to milliseconds. While this millisecond-scale lifetime is aligned with the triplet state lifetime of NCA molecules, the emission at 460 nm differs from the typical phosphorescence wavelength of NCA triplets, which occurs at longer wavelengths.[12,13] This observation, combined with the appropriate energy level alignment shown in Figure 1a, supports the interpretation that the delayed emission originates from QD repopulated through rTET from NCA triplets.

PL lifetime curves fitting

Multi-exponential functions were used to fit the PL and TADPL lifetime curves of the QDs:

$$I(t) = A_0 + \sum_i A_i e^{-\frac{t}{\tau_i}} \tag{S11}$$

Where $I(t)$ is the PL intensity at decay time t, τ_i and A_i are the lifetime and the corresponding amplitude. A_0 is the offset. The average lifetime $\langle\tau\rangle$ was calculated as:

$$\langle\tau\rangle = \frac{\sum_i A_i \tau_i^2}{\sum_i A_i \tau_i} \tag{S12}$$

All the fitting results are shown in Table S2.

Table S2. Fitting parameters for PL lifetime curves

QD

Temperature (K)	A1	t1 (ns)	A2	t2 (ns)	A3	t3 (ns)	< τ > (ns)
193	1.117	6.089	0.014	15.332	-	-	6.372
213	1.038	6.781	0.016	24.605	-	-	7.725
233	1.047	7.194	0.0124	39.673	-	-	9.185
253	1.045	7.534	0.0115	41.849	-	-	9.511
273	1.061	7.691	0.0122	44.724	-	-	10.012
293	1.046	8.027	0.0127	49.244	-	-	10.884

QD-NCA

Temperature (K)	A1	t1 (ms)	A2	t2 (ms)	A3	t3 (ms)	< τ > (ms)
193	2.685	0.867	0.151	7.984	0.019	88.542	30.791
213	2.472	0.927	0.153	7.574	0.024	69.838	24.960
233	2.356	0.975	0.138	6.863	0.036	50.15	19.661
253	2.173	1.059	0.129	6.668	0.046	39.413	16.008
273	2.209	1.002	0.19	6.168	0.035	38.413	12.916
293	3.272	0.74	0.143	5.722	0.032	36.663	11.215

SI8: Fitting of Temperature Dependent Bandgap of QDs

Varshni model fitting

The Varshni empirical equation has been widely used to describe the temperature dependence of the energy gap in semiconductor:[14]

$$E_g(T) = E_g(0) - \frac{\alpha T^2}{T + \beta} \tag{S13}$$

Where $E_g(T)$ represents the band gap energy at T K, $E_g(0)$ is the band gap energy at 0 K, α is the temperature coefficient, and β is the parameter related to Debye temperature. According to Figure 4a, the fitting yields: $E_g(0)$ = (2.758 ± 0.003) eV, α = (1.58 ± 0.28) × 10^{-4} eV/K and β = (197 ± 57) K. The relatively small value of α indicates that the thermal stability of the CdSe core has been significantly enhanced due to the ZnS cladding.

References

1 Wang, L.; Mu, P.; Zhou, Z.; Zhang, X.; Liu, D.; Liang, Y.; Liang, G. Ultrafast Spectroscopy of Size-Dependent Hole-Transfer-Mediated Triplet Energy Transfer in a Quantum Dot-Molecule Complex. *Results Phys.* **2023**, *55*, 107150.

2 Mongin, C.; Moroz, P.; Zamkov, M.; Castellano, F. N. Thermally Activated Delayed Photoluminescence from Pyrenyl-Functionalized CdSe Quantum Dots. *Nat. Chem.* **2018**, *10*, 225-230.

3 Yin, H.; Li, H.; Xia, G.; Ruan, C.; Shi, Y.; Wang, H.; Jin, M.; Ding, D. A Novel Non-Fluorescent Excited State Intramolecular Proton Transfer Phenomenon Induced by Intramolecular Hydrogen Bonds: An Experimental and Theoretical Investigation. *Sci. Rep.* **2016**, *6*, 19774.

4 Li, B.; Li, Q.; Zhu, L.; Li, K.; Lin, F.; Shi, Y.; Wei, Z. Enhancing Förster Resonance Energy-Transfer Rates in Perovskite Quantum Dots via Controlling Donor–Acceptor Ratios. *J. Phys. Chem. C* **2024**, *128*, 8716-8723.

5 Brus, L. E. Electron–Electron and Electron-Hole Interactions in Small Semiconductor Crystallites: The Size Dependence of the Lowest Excited Electronic State. *J. Chem. Phys.* **1984**, *80*, 4403-4409.

6 Brus, L. Electronic Wave Functions in Semiconductor Clusters: Experiment and Theory. *J. Phys. Chem.* **1986**, *90*, 2555-2560.

7 Zhang, X.; Chen, L.; Yuan, L.; Liu, R.; Li, D.; Liu, X.; Ge, G. Conformation-Dependent Coordination of Carboxylic Acids with Fe3 O4 Nanoparticles Studied by ATR-FTIR Spectral Deconvolution. Langmuir 2019, 35, 5770-5778. https://doi.org/10.1021/acs.langmuir.8b03303.

8 Świderski, G.; Świsłocka, R.; Łyszczek, R.; Wojtulewski, S.; Samsonowicz, M.; Lewandowski, W. Thermal, Spectroscopic, X-Ray and Theoretical Studies of Metal Complexes (Sodium, Manganese, Copper, Nickel, Cobalt and Zinc) with Pyrimidine-5-Carboxylic and Pyrimidine-2-Carboxylic Acids. J. Therm. Anal. Calorim. 2019, 138, 2813-2837. https://doi.org/10.1007/s10973-019-08594-x.

9 Mongin, C.; Garakyaraghi, S.; Razgoniaeva, N.; Zamkov, M.; Castellano, F. N. Direct Observation of Triplet Energy Transfer from Semiconductor Nanocrystals. *Science* **2016**, *351*, 369-372.

10 Luo, X.; Lai, R.; Li, Y.; Han, Y.; Liang, G.; Liu, X.; Ding, T.; Wang, J.; Wu, K. Triplet Energy Transfer from $CsPbBr_3$ Nanocrystals Enabled by Quantum Confinement. *J. Am. Chem. Soc.* **2019**, *141*, 4186-4190.

11 Anderson, A. Y.; Barnes, P. R. F.; Durrant, J. R.; O'Regan, B. C. Quantifying Regeneration in Dye-Sensitized Solar Cells. *J. Phys. Chem. C* **2011**, *115*, 2439-2447.

12 Zhang, X.; Hudson, M. H.; Castellano, F. N. Engineering Long-Lived Blue Photoluminescence from InP Quantum Dots Using Isomers of Naphthoic Acid. J.

Am. Chem. Soc. 2022, 144, 3527-3534.

13 Cossairt, B. M.; Juhas, P.; Billinge, S. J. L.; Owen, J. S. Tuning the Surface Structure and Optical Properties of CdSe Clusters Using Coordination Chemistry. J. Phys. Chem. Lett. 2011, 2, 3075-3080.

14 Varshni, Y. P. Temperature Dependence of the Energy Gap in Semiconductors. *Physica* **1967**, *34*, 149-154.
